# A Novel Fluorescent Reporter System Identifies Laminin-511/521 as Potent Regulators of Cardiomyocyte Maturation

**DOI:** 10.1038/s41598-020-61163-3

**Published:** 2020-03-06

**Authors:** Nawin Chanthra, Tomoyuki Abe, Matthew Miyamoto, Kiyotoshi Sekiguchi, Chulan Kwon, Yutaka Hanazono, Hideki Uosaki

**Affiliations:** 1 Division of Regenerative Medicine, Center for Molecular Medicine, Jichi Medical University, Shimotsuke, Japan; 2 Division of Cardiology, Johns Hopkins University School of Medicine, Baltimore, USA; 3 Institute for Cell Engineering, Johns Hopkins University School of Medicine, Baltimore, USA; 4 Division of Matrixome Research and Application, Institute for Protein Research, Osaka University, Osaka, Japan; 5 Division of Stem Cell Research and Drug Development, Center for Development of Advanced Medical Technology, Jichi Medical University, Shimotsuke, Japan

**Keywords:** Developmental biology, Pluripotent stem cells, Cardiovascular biology, Assay systems, Extracellular matrix

## Abstract

Pluripotent stem cell-derived cardiomyocytes (PSC-CMs) hold great promise for disease modeling and drug discovery. However, PSC-CMs exhibit immature phenotypes in culture, and the lack of maturity limits their broad applications. While physical and functional analyses are generally used to determine the status of cardiomyocyte maturation, they could be time-consuming and often present challenges in comparing maturation-enhancing strategies. Therefore, there is a demand for a method to assess cardiomyocyte maturation rapidly and reproducibly. In this study, we found that *Myomesin-2* (*Myom2*), encoding M-protein, is upregulated postnatally, and based on this, we targeted TagRFP to the *Myom2* locus in mouse embryonic stem cells. Myom2-RFP^+^ PSC-CMs exhibited more mature phenotypes than RFP^-^ cells in morphology, function and transcriptionally, conductive to sarcomere shortening assays. Using this system, we screened extracellular matrices (ECMs) and identified laminin-511/521 as potent enhancers of cardiomyocyte maturation. Together, we developed and characterized a novel fluorescent reporter system for the assessment of cardiomyocyte maturation and identified potent maturation-enhancing ECMs through this simple and rapid assay. This system is expected to facilitate use of PSC-CMs in a variety of scientific and medical investigations.

## Introduction

Pluripotent stem cell-derived cardiomyocytes (PSC-CMs) provide an unlimited source of cardiomyocytes for disease modeling and drug screening^1–4^, bypassing the need for using primary cardiomyocytes obtained from patients, which are difficult to maintain in a dish^5^. Directed cardiac differentiation protocols have been developed by sequential addition of growth factors or small molecules, resulting in the efficient production of PSC-CMs^6–9^. However, these PSC-CMs remain largely immature as fetal or neonatal cardiomyocytes with regards to their structure and function, never undergoing typical *in vivo* postnatal cardiomyocyte maturation^10–13^. These immature phenotypes of PSC-CMs lead to a failure of recapitulating the disease pathology as presented in mature cardiomyocytes, resulting in an inaccurate prediction of drug response that being tested^14,15^. This limitation hampers the utilities of PSC-CMs for medical purposes, especially for *in-vitro* models and drug discovery. Thus, there is a critical demand for sophisticated strategies to enhance the maturation of PSC-CMs.

Over the last decade, there have been enormous efforts to develop maturation strategies for PSC-CMs. For instance, PSC-CMs that were cultured on extracellular matrices (ECMs), which are essential in transmitting signals between cardiomyocytes and neighboring tissues, significantly improved their electrophysiological and structural maturation, or increased expressions of cardiac genes^16,17^. Additionally, hormone treatments are also considered to promote cardiomyocyte maturation^18^. In these studies, structural and functional assays are broadly used to determine the effects of those strategies on cardiomyocyte maturation. These methods range from immunostaining for cell morphology and sarcomere structure^19^, transcriptional analysis for global gene expressions^12^, physiological experiment for action potential^20^ and calcium handling^21^, assessment of contractile forces^22^, and measurement of mitochondrial activity^23^. However, these assays vary from lab-to-lab and are time-consuming and difficult to compare between maturation strategies. To date, an appropriate high-throughput method to assess PSC-CM maturation has not yet been established.

Fluorescent reporters have been widely used to understand and improve differentiation of a certain cell type or lineage in developmental biology and stem cell biology^24^. Thus, we hypothesized that a novel fluorescent reporter line would help determine the maturation state of PSC-CMs easily and rapidly. Here, we developed a novel fluorescent reporter line, using *Myom2* gene as a candidate maker of cardiomyocyte maturation. Using this reporter line, we screened a wide range of ECMs including laminin, collagen, and fibronectin. We found that laminin-511/521 E8 fragments are the most powerful ECM enhancers of cardiomyocyte maturation. These findings suggest that our maturation reporter line will be effective in optimizing robust pro-maturation culture conditions, and will help unlock their potential in disease modeling and drug screening.

## Results

### Generation of a fluorescent reporter line for cardiomyocyte maturation

**Figure 1:**
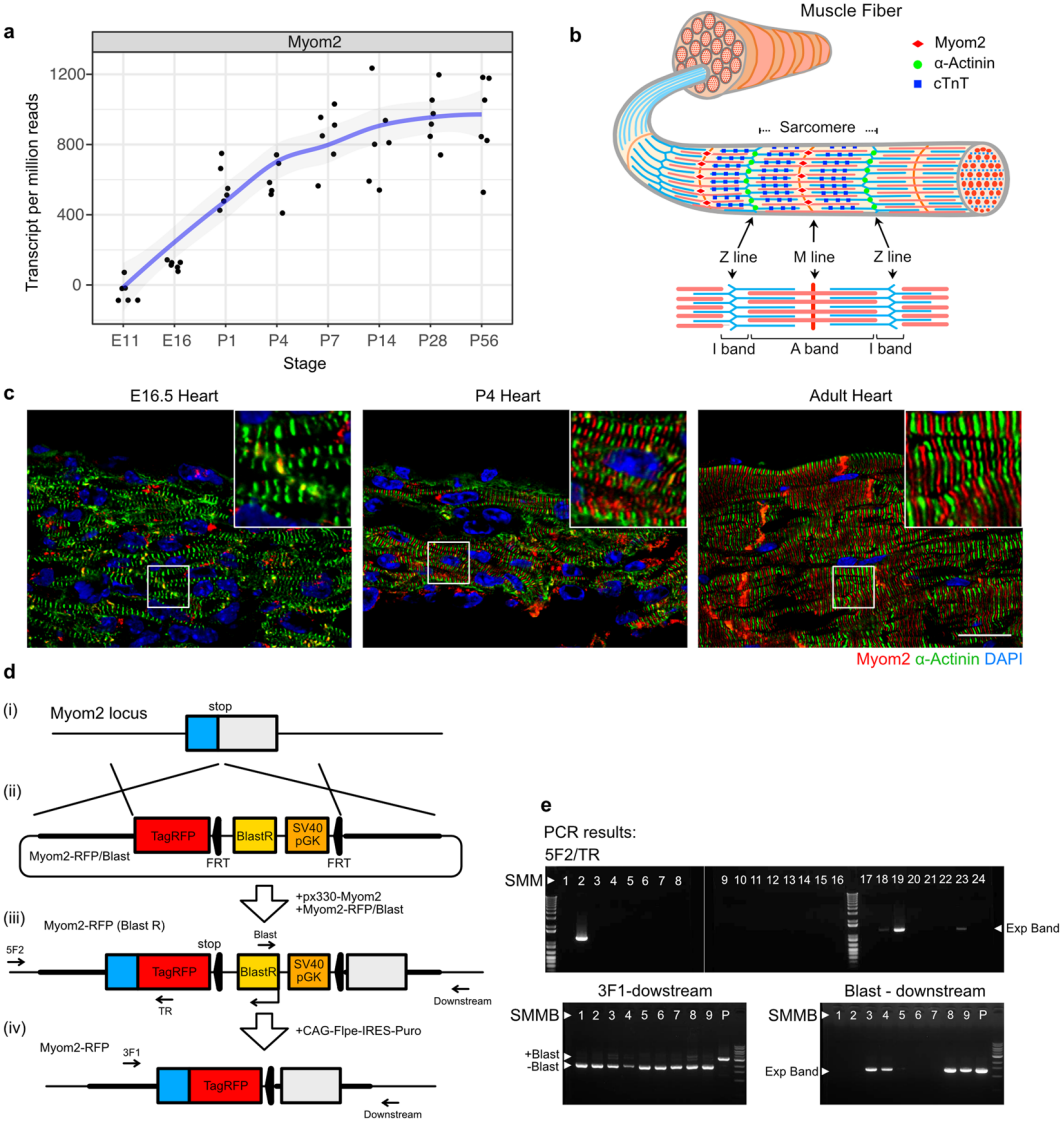
CRISPR/Cas9-mediated knock-in of TagRFP into *Myom2*. (**a**) Myom2 expression during heart development (from embryonic day (E) 11 to postnatal day (P) 56). 3′ mRNA-seq results are shown as transcripts per million reads. A blue line indicates local regression curve using locally estimated scatterplot smoothing (LOESS). (**b**) A scheme showing localizations of Myom2 in M-line, α-actinin in Z-line, and cardiac troponin T (cTnT) in actin thin filaments of sarcomere structure. (**c**) Representative images of Myom2 expression and localization in different stages of heart development including E16.5, P4, and adult heart; Myom2 (red), α-actinin (green), and DAPI for nuclei (blue). Higher magnification images are shown in insets. Scale bar, 20 μm. (**d**) A schematic overview of (i) wild-type *Myom2* locus, (ii) Myom2-RFP/Blast targeting construct, knocked-in Myom2-RFP (iii) with and (iv) without a blasticidin-resistance cassette, and forward and reverse primer binding sites for testing knock-in. (**e**) PCR screen to identify correct targeted clones: 3′ end screen of integration of TagRFP in subclones SMM2, 18, 19, and 23 (Top panel). Confirmation of deletion of the blasticidin-resistance cassette in subclones SMMB1, 2, 5, 6, and 7 (Bottom panels, both left and right).

Fluorescent reporters are often used in developmental and stem cell biology for studying differentiation and biological processes. Thus, we hypothesized that a new fluorescent reporter for cardiomyocyte maturation would serve as an effective tool to study cardiomyocyte maturation *in vitro*. Consistent with previous studies, we found that expression of *Myom2*, encoding Myomesin-2 (Myom2, also known as M-protein) exclusively localized to M-line of sarcomeres, is upregulated in neonatal heart, and continuously increases to adult (Fig. 1a–c)^12,25–28^. Myom2 is strongly conserved among chicken, mouse, and human^25^. Moreover, while PSC-CMs remain immature and display fetal features^12^, Myom2 protein became detectable along sarcomere postnatally (Fig. 1c). These data supported Myom2 as an attractive candidate for development as a cardiomyocyte maturation reporter.

Myom2 is not only expressed in cardiac muscle but also found in skeletal muscle^25^. Thus, purification of cardiomyocytes *in vitro* is required. To this end, we used a mouse embryonic stem cell (mESC) line named syNP4, which has a puromycin resistance cassette driven by sodium-calcium exchanger 1 (*NCX1*) promoter^6^, as the parental mESCs. *NCX1* promoter is active in cardiomyocytes, but inactive in other cell types^29^. Therefore, we could selectively obtain cardiomyocytes by puromycin treatment. To generate a reporter-line, we inserted TagRFP to 3′ end of *Myom2* genomic locus by transfecting a vector expressing Cas9 and single-guide RNA and a targeting construct (Fig. 1d). TagRFP knocking-in to *Myom2* allows us to monitor the expression level and localization of Myom2 as TagRFP fuses to C-terminal of Myom2. After blasticidin selection, the insertion of TagRFP into *Myom2* genomic locus was confirmed in several subclones including SMM2, 18, 19, and 23 (Fig. 1e, top panel). After confirming that SMM18 differentiated to cardiomyocytes similarly to the parental line syNP4, the blasticidin-resistance cassette was removed from SMM18 using flippase site-specific recombination (Fig. 1d). Correct targeting of the subclones and excision of the blasticidin-resistance cassette were confirmed by PCR and sequencing (Fig. 1e, bottom panel, data not shown). The result demonstrated that SMMB1, 2, 5, 6, and 7 were correctly targeted with TagRFP and excised the blasticidin-resistance cassette. We further confirmed that SMMB2 and 5 differentiated well to cardiomyocytes. In this study, we used SMM18 and SMMB2 for the following experiments.

### RFP^+^ cardiomyocytes are more mature than RFP^−^ cardiomyocytes

**Figure 2:**
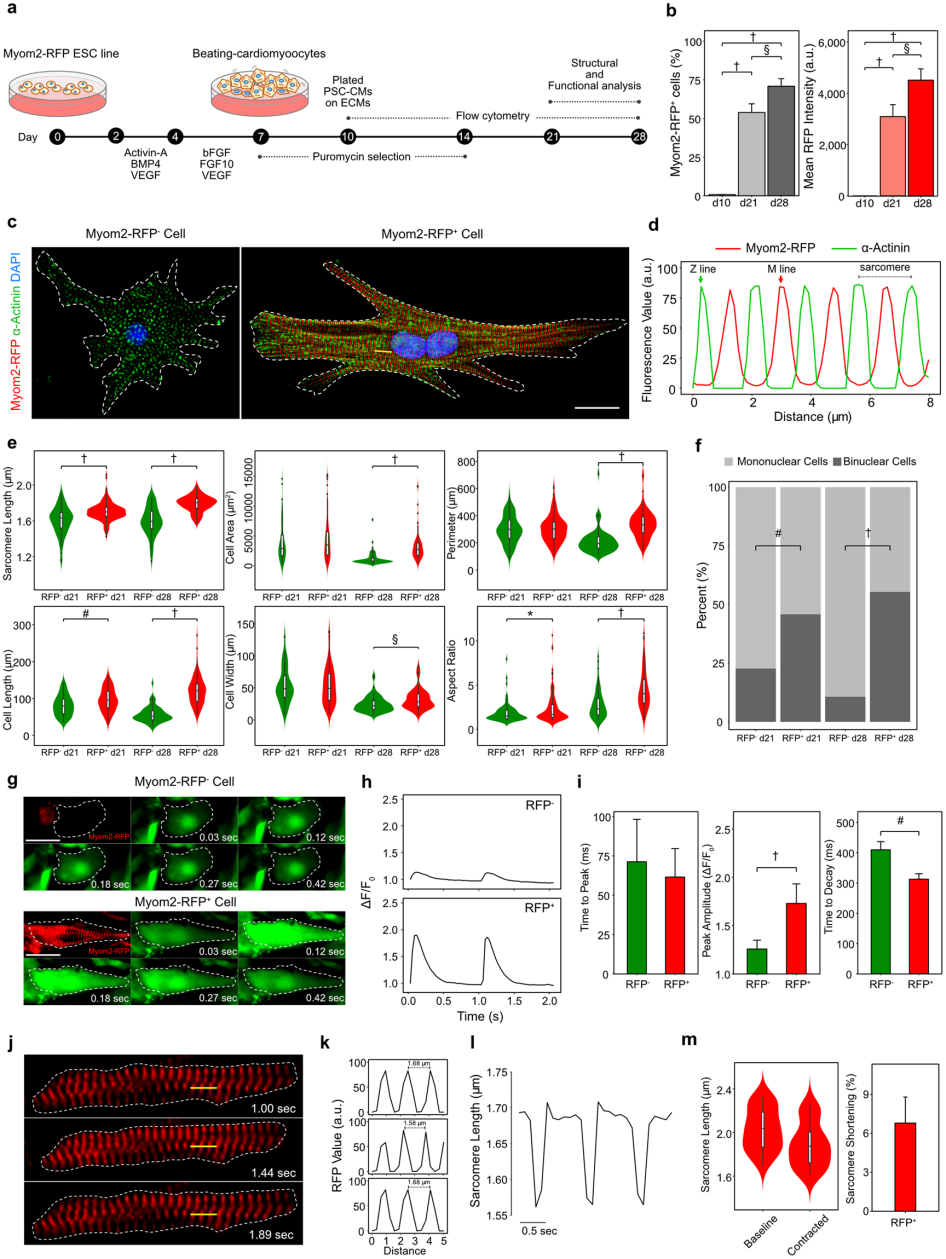
Characterizations of the Myom2-RFP ESC line as a cardiomyocyte maturation reporter. (**a**) A schematic protocol for cardiac differentiation. (**b**) Myom2-RFP expression during a prolonged culture. Data is presented as means ± SD (*n* = 4). One-way ANOVA with post-hoc Tukey HSD test; § *P* < 0.01, † *P* < 0.0001. Fluorescence intensity is presented as arbitrary unit (a.u.). (**c**) Representative fluorescence images of RFP^−^ and RFP^+^ cardiomyocytes at day 28. A yellow line indicates the line scan region displayed in (**d**). Myom2-RFP (red); α-actinin (green); Nuclei (blue). Scale bar, 20 μm. (**d**) Corresponding line scan for the altered pattern of Myom2-RFP and α-actinin in a sarcomere. (**e**) Comparisons of structure and morphology between RFP^−^ and RFP^+^ cardiomyocytes (*n* > 65). Sarcomere length, cell area, perimeter length, cell length, cell width, and aspect ratio were examined. For violin plots, black lines in the white boxes show the medians, box limits indicate the 25th and 75th percentiles, whiskers extend 1.5 times the interquartile range from the 25th and 75th percentiles, and polygons represent density estimates of data and extend to extreme values. Student’s *t* test; *P < 0.05, ^§^*P* < 0.01, ^#^*P* < 0.001, ^†^*P < *0.0001. (**f**) Proportion of mononuclear and binuclear cells in RF*P*^+^ and RFP^−^ PSC-CMs at day 21 and 28 (n > 65). Chi-square Test; ^#^*P* < 0.001, ^†^*P* < 0.0001. (**g–i**) Calcium transients of RFP^−^ and RFP^+^ cardiomyocytes: (**g**) Representative time-lapse images of calcium transients for RFP^−^ and RFP^+^ cardiomyocytes (scale bar, 20 μm), and (**h**) corresponding spatially averaged profiles of cytosolic calcium transients evoked by electrical field stimulation at 1 Hz, at day 28. (**i**) Mean values of time to peak, peak amplitude, and decay time of Ca^2+^ transients (*n* > 30). Student’s *t* test; ^#^*P* < 0.001, ^†^*P < *0.0001. (**j–m**) Sarcomere shortening assays for PSC-CMs using Myom2-RFP: (**j**) Representative images for sarcomere shortening with measuring regions as indicated by yellow bars and (**k**) corresponding line scans of sarcomere lengths. (**l**) Sarcomere shortening profile of RFP^+^ cardiomyocytes. (**m**) Normalized sarcomere lengths and percent of sarcomere shortening of RFP^+^ cardiomyocytes (n = 38).

SMM18 or SMMB2 were differentiated to PSC-CMs by sequential addition of cytokines as described previously^12^. PSC-CMs were then purified with puromycin selection. Structural and functional analyses were conducted up to day 28 of differentiation (Fig. 2a). First, we profiled RFP expression over the course of a prolonged culture, that is known to enhance cardiomyocyte maturation *in vitro*^30^. We examined both the proportion of cells expressing Myom2-RFP (RFP^+^) in PSC-CMs and the intensity of RFP in RFP^+^ cells using flow cytometry. Myom2-RFP was not detected immediately after 10 days of differentiation (less than 1%), whereas a prolonged culture from day 10 to day 28 resulted in significant increases of both the proportion of RFP^+^ cardiomyocytes and RFP intensity (Fig. 2b). These results supported our hypothesis that Myom2-RFP could be useful to examine cardiomyocyte maturation.

Despite the proportion of RFP^+^ cardiomyocytes increasing during the prolonged culture, PSC-CMs generated from the reporter line yielded a heterogeneous population of RFP^+^ (more than 70%) and RFP^−^ (about 30%) cardiomyocytes at day 28. To validate whether RFP^+^ PSC-CMs are indeed more mature than RFP^−^ ones, we examined their structure, morphology, binucleation, and physiology of RFP^+^ and RFP^−^ cells at days 21 and 28. In agreement with the Myom2 localization to M-line (Fig. 1b,c), parallel alignment of M-line and the altered pattern with α-actinin in Z-line along the cells were clearly observed in RFP^+^ cardiomyocytes, but not in RFP^−^ cardiomyocytes (Fig. 2c). Representative line scan for altered pattern of Myom2-RFP and α-actinin is presented in Fig. 2d. Troponin T that localize on actin filament showed an exclusive pattern with RFP, solidified the finding of RFP localizing to M-line (Supplementary Fig. S1). In contrast to the well-aligned sarcomeres in RFP^+^ PSC-CMs, those in RFP^−^ cells were disarrayed, suggesting that RFP^+^ PSC-CMs were more mature than RFP^−^ cells (Fig. 2c; Supplementary Fig. S2). Next, we further examined fluorescence images. RFP^+^ PSC-CMs had longer sarcomere length, increased cell size, and perimeter, as well as improved geometry (cell length, cell width, and aspect ratio) toward adult-like cardiomyocytes, especially at day 28, compared to RFP^−^ cardiomyocytes (Fig. 2e). We also found that RFP^+^ cardiomyocytes showed a higher binuclear cell population than RFP^−^ cardiomyocytes (Fig. 2f). These results demonstrated that RFP^+^ cardiomyocytes displayed structurally and morphologically more mature phenotypes than RFP^−^ cardiomyocytes.

In addition to structural and morphological analysis, we also examined a physiological feature by measuring calcium transients in RFP^−^ and RFP^+^ cardiomyocytes at day 28 of cardiac differentiation. Calbryte 520-AM, a calcium-sensing dye, was loaded to PSC-CMs and calcium transients were obtained using time-lapse imaging, while PSC-CMs were evoked by an electrical-field stimulation at 1 Hz (Fig. 2g). Time-lapse images of each PSC-CM were examined and the trace of calcium transient was obtained from the entire cell. Calcium transient of RFP^+^ PSC-CMs showed a higher amplitude and faster decay time to baseline than that of RFP^−^ cardiomyocytes (Fig. 2h,i). Moreover, we could obtain sarcomere shortening, which is hardly observed in PSC-CMs, with time-lapse images detecting RFP during cardiomyocyte contraction (Fig. 2j–m). RFP^+^ cardiomyocytes displayed constant contraction, and sarcomere shortening was approximately 6.8% (from about 2.03 ± 0.19 μm down to 1.89 ± 0.20 μm) (Fig. 2l,m). Taken together, RFP^+^ cardiomyocytes were morphologically, structurally and functionally more mature than RFP^−^ cardiomyocytes, and the Myom2-RFP ESC line can be used as a cardiomyocyte maturation reporter.

### RFP^+^ PSC-CMs are transcriptionally more mature than RFP^−^ cardiomyocytes

**Figure 3:**
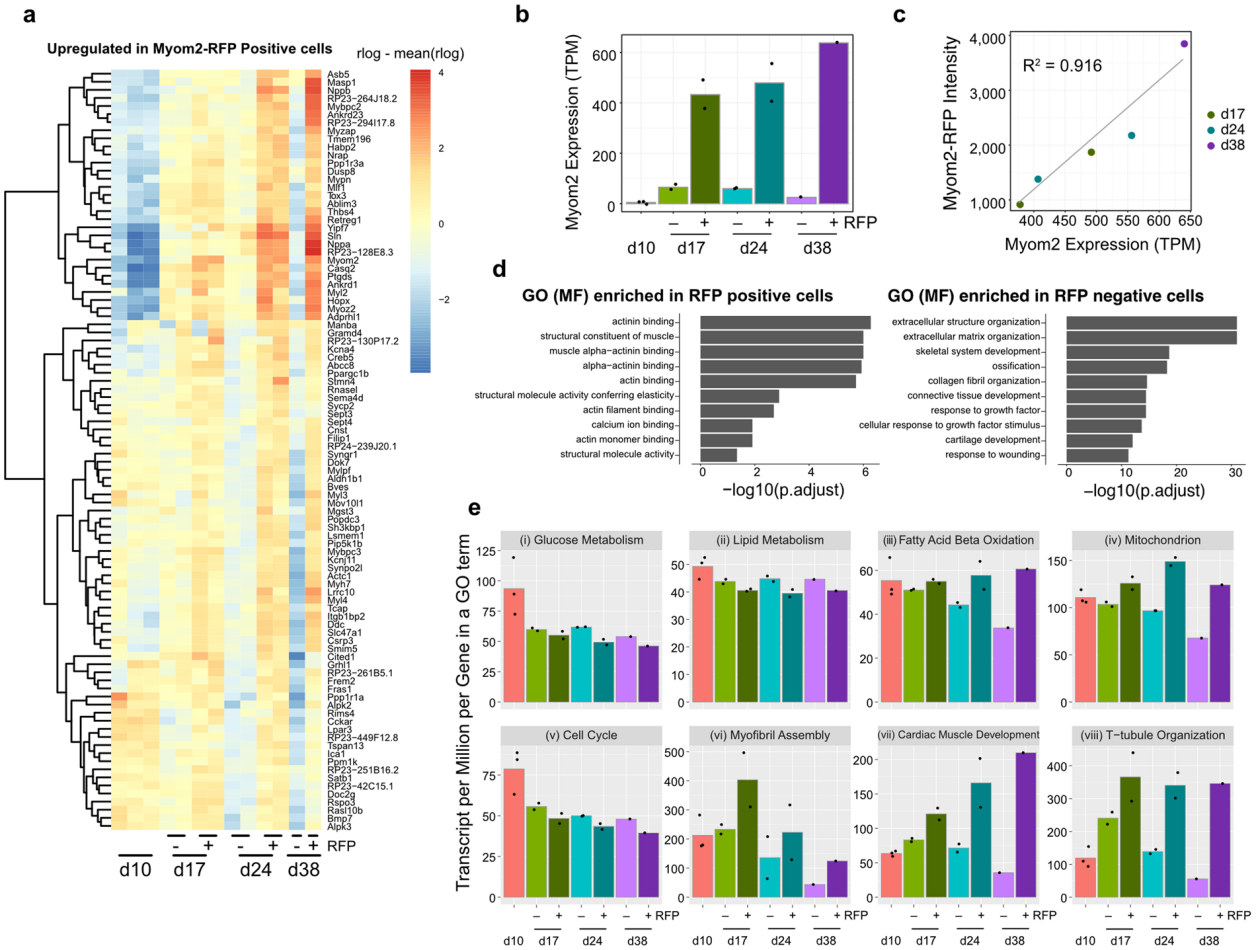
Transcriptome analysis of RFP^+^ and RFP^−^ cardiomyocytes. (**a**) A heatmap of upregulated genes in RFP^+^ PSC-CMs at days 17 and 24 compared to RFP^−^ cells. Rlog values are coded on the red-to-blue scale (higher expression, red; lower expression, blue). (**b**) Expressions of Myom2 in PSC-CMs at different time points (day 10, 17, 24, and 38). (**c**) Correlation between Myom2 expressions and RFP intensities in RFP^+^ PSC-CMs from day 17 to day 38. The correlation coefficient (R2) between Myom2 expression levels and RFP intensities was 0.916. (**d**) GO terms for molecular function identify for differentially enriched genes in RFP^+^ and RFP^−^ cardiomyocytes. The x-axis represents the logarithms of the adjusted *P*-value. (**e**) Averaged gene expression of 8 selected GO terms for biological processes.

To further confirm if RFP^+^ PSC-CMs are more mature than RFP^−^ cells, we performed RNA-sequencing. To this end, we sorted RFP^+^ and RFP^−^ cardiomyocytes at 17, 24, and 38 days of cardiac differentiation using a cell-sorter. Then, the sorted-cells and PSC-CMs at day 10, were subjected to RNA sequencing. Genes enriched in RFP^+^ cells compared to RFP^−^ ones at days 17, 24, and 38 are shown in Fig. 3a. Known genes related to cardiomyocyte maturation were upregulated in RFP^+^ cardiomyocytes such as sarcomere genes (*Myh7*, *Myl2*, *Myl3*, *Myoz2*, and *Mypn*), calcium-handling gene (*Casq2*), and ion transporter at sarcolemma (*Kcna4*) (Fig. 3a). To note, Myom2 expression was highly enriched in RFP^+^ cardiomyocytes, and Myom2 expression levels and Myom2-RFP intensity in RFP^+^ cells had a strong correlation (R^2^ = 0.916; Fig. 3b,c). Gene ontology (GO) analysis revealed that GO terms involved in structural and muscle development are enriched in RFP^+^ cardiomyocytes, whereas GO terms of ECM organization were highly enriched in RFP^−^ cardiomyocytes (Fig. 3d). To explore the functional characteristics of the differentially expressed genes, we examined overall gene expression levels in selected GO terms of biological processes (Fig. 3e)^31^. Interestingly, genes in glucose metabolic process and cell cycle, both related to immature cardiomyocyte state, were downregulated soon after cardiac differentiation at day 10 in both RFP^+^ and RFP^−^ cells, although they were slightly more downregulated in RFP^+^ PSC-CMs (Fig. 3e-i,v). As consistent with the notion above, genes in fatty acid β-oxidation and mitochondrion were upregulated in RFP^ +^ cardiomyocytes compared to RFP^−^ cardiomyocytes, suggesting that a metabolic switch was occurring in RFP^+^ cardiomyocytes, which occurs postnatally *in vivo* (Fig. 3e-ii-iv). Moreover, Genes in myofibril assembly, cardiac muscle tissue development, and T-tubule organization were upregulated in RFP^+^ cardiomyocytes (Fig. 3e-vi-viii). These results confirmed that RFP^+^ cardiomyocytes were transcriptionally more mature than RFP^−^ cardiomyocytes.

### Laminin-511/521 E8 fragments increase Myom2-RFP intensity and structural maturation of PSC-CMs

**Figure 4:**
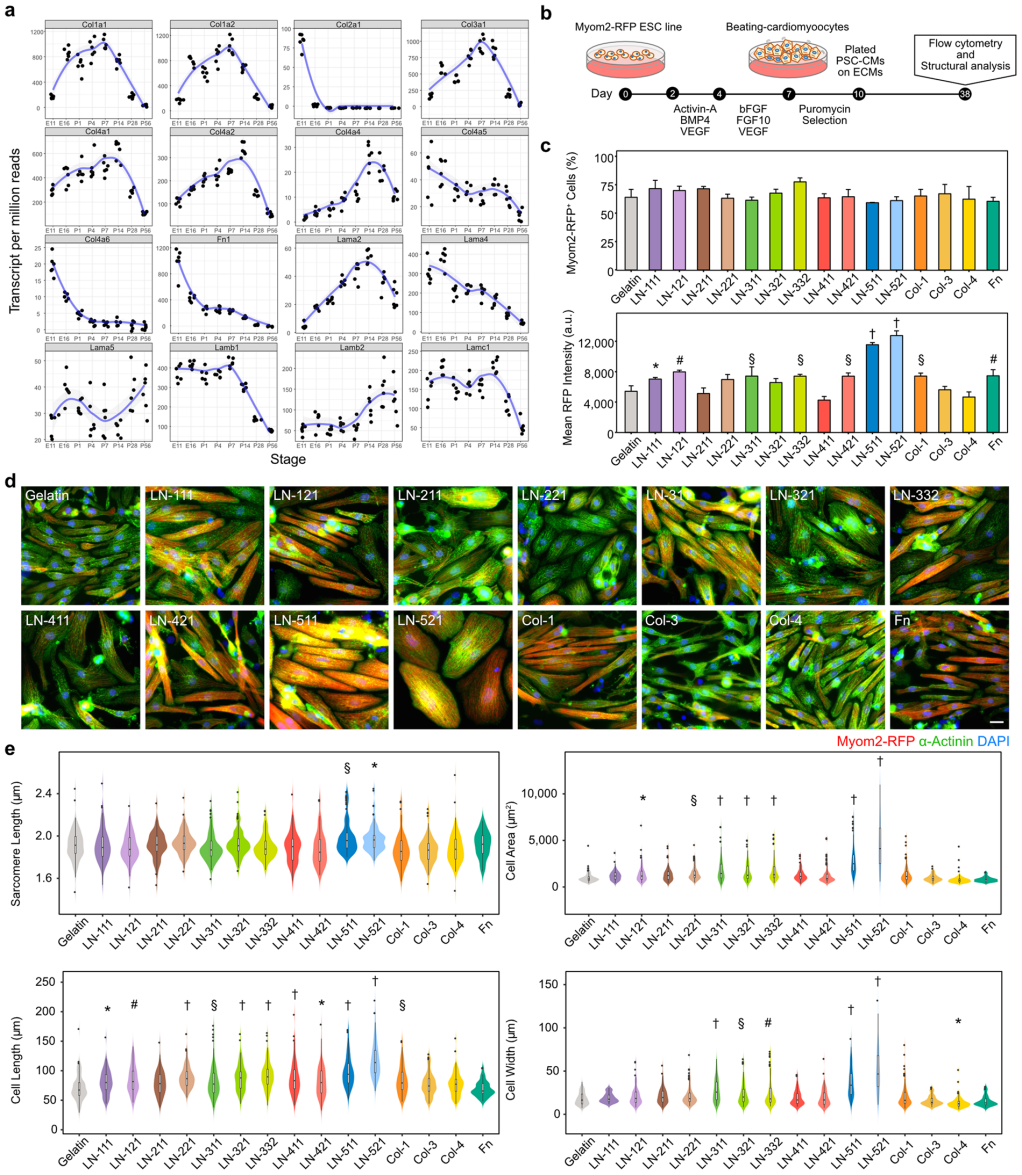
ECMs increased RFP intensity and led to structural maturation. (**a**) Expression profiles of selected ECMs including laminin, collagen, and fibronectin during heart development (E11 to P56). (**b**) A schematic diagram of the experiment for quantification of Myom2-RFP expression and structural analysis after ECM treatments for 38 days of cardiac differentiation. (**c**) Effects of ECM treatments on fraction of RFP^+^ cardiomyocytes and RFP intensity. Data are presented as means ± SD (*n* = 3). Fluorescence intensities are presented as arbitrary unit (a.u.). Dunnett’s test was used to compare each ECM treatment with a control (gelatin); *P < 0.05, ^§^*P* < 0.01, ^#^*P* < 0.001, ^†^*P* < 0.0001. (**d**) Representative images for ECM-treated cells stained with α-actinin antibody (green), TagRFP (red), and DAPI for nuclei (blue). Scale bar, 20 μm. (**e**) Statistics of structural and morphological features. Sarcomere length, cell area, cell length, and cell width were examined. To note, ECM-treated cells exhibited significant increase in sarcomere length, cell area, cell length, and cell width, especially with laminin (LN)-511/521 treatments, compared with controls. Violin plots are explained in Fig. 2. *n* > 100 from three different cardiac differe*n*tiation runs. Dunnett’s test was used to compare each ECM treatment with a control (gelatin); *P < 0.05, ^§^*P* < 0.01, ^#^*P* < 0.001, ^†^*P < *0.0001.

To test if Myom2-RFP could be used for screening purposes of cardiomyocyte maturation, we examined the effects of ECMs on PSC-CM maturation. ECMs, containing important signaling molecules and providing structural support, are critical for heart development. To identify candidate ECMs that promote cardiomyocyte maturation, we performed RNA-sequencing of wild-type mouse ventricles ranging from embryonic day 11 (E11) to postnatal day 56 (P56) (Supplementary Fig. S3). The hierarchical clustering illustrated groupings of the gene expression patterns among early embryonic, neonatal, and adult ventricles (Supplementary Fig. S3). Interestingly, fibronectin (*Fn1*), and collagen type II (*Col2a1*) and type IV (*Col4a6*), were abundantly expressed in embryonic hearts, whereas collagen type I (*Col1a1* and *Col1a2*) and III (*Col3a1*) were highly upregulated in neonatal hearts (Fig. 4a and Supplementary Fig. S3). Furthermore, laminin (α2, α5, and β2) and one of collagen type IV members (*Col4a4*) were expressed later in adult hearts (Fig. 4a and Supplementary Fig. S3).

To evaluate the effects of ECMs on cardiomyocyte maturation, we plated PSC-CMs on various concentrations (ranging from 0.125 to 1 μg/cm^2^) of laminin E8 fragments (isoform 111, 121, 211, 221, 311, 321, 332, 411, 421, 511, and 521)^32^, collagen (type I, III, and IV), and fibronectin at day 10 of differentiation (Fig. 4b). To determine RFP^+^ cardiomyocytes and RFP intensity quantitatively, flow-cytometry was performed after culturing PSC-CMs on the ECMs for 7, 14, and 28 days (17 to 38 days after differentiation). We found that both the proportion of RFP^+^ cardiomyocytes and the RFP intensity increased from day 17 to 38 (Supplementary Fig. S4). We observed the increased RFP intensity in PSC-CMs on some ECMs with a dose-dependent manner (Supplementary Fig. S4). The results of 1 μg/cm^2^ of ECMs at day 38 of differentiation were summarized in Fig. 4c. Among ECMs tested, laminin-511/521 showed the highest RFP intensity compared to others (Fig. 4c). However, the proportion of RFP^+^ cardiomyocytes remained the same (Fig. 4c). This result implicated that ECMs, especially laminin-511/521 enhanced cardiomyocyte maturation *in vitro* rather than initiating maturation.

Next, we examined the structural and morphological features of PSC-CMs on ECMs (Fig. 4d,e). We found that control cells (plated on gelatin) were small, while PSC-CMs cultured on laminin-511/521 showed significantly increased cell size, cell length, and cell width (Fig. 4e). Moreover, the sarcomere lengths of the PSC-CMs were longer on laminin-511/521 compared to control (Fig. 4e). This result indicated that laminin-511/521 robustly promoted the structural maturation of cardiomyocytes.

### Laminin-511/521 E8 fragments enhance functional maturation of PSC-CMs

**Figure 5:**
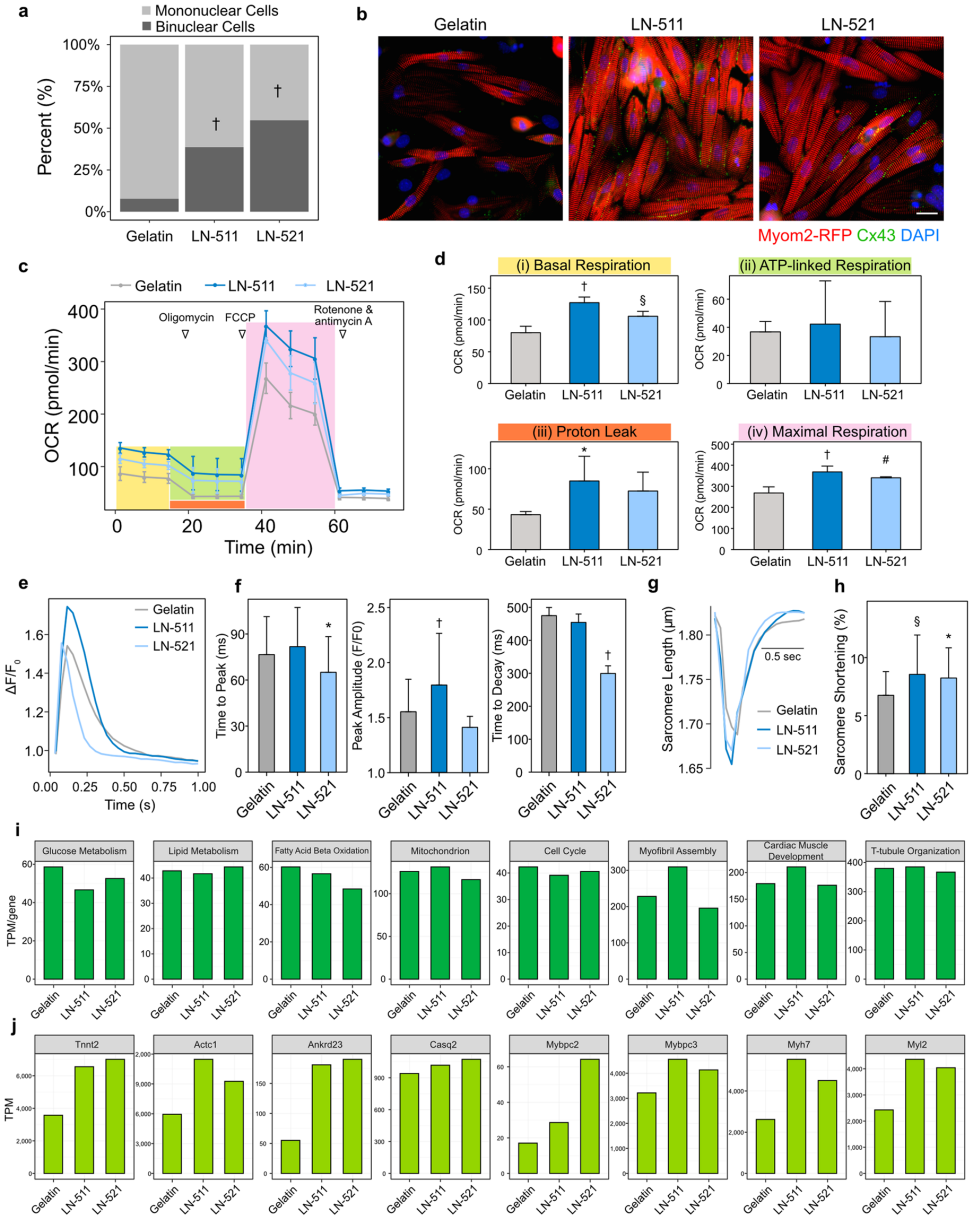
Functional and transcriptional analysis after laminin-511/521 treatments. (**a**) Proportions of mononuclear and binuclear cells in PSC-CMs cultured on laminin-511/521 compared to gelatin (*n* > 100). (**b**) Representative images of PSC-CMs stained for Cx43 at day 38 of differentiation. Scale bar, 20 μm. (**c**) Representative traces for PSC-CMs on laminin-511/521 and gelatin responding to oligomycin, FCCP, and rotenone/antimycin A. (**d**) Statistical analyses of OCRs for (i) Basal respiration, (ii) ATP-linked respiration, (iii) proton leak, and (iv) maximal respiration. Data are presented as means ± SD (*n* = 3). Dunnett’s test was used to compare PSC-CMs on laminin with ones on gelatin as a control; *P < 0.05, ^§^*P* < 0.01, ^#^*P* < 0.001, ^†^*P* < 0.0001. (**e**) Representative intracellular calcium transients from PSC-CMs on gelatin and laminin-511/521, stimulated at 1 Hz, at day 38. (**f**) Mean values of time to peak, peak amplitude, and decay time of Ca^2+^ transients (*n* > 80). Dunnett’s test; *P < 0.05, ^†^*P < *0.0001. (**g**) Representative traces showing sarcomere length during electric-pulse stimulation (1 Hz). (**h**) Averaged data of sarcomere shortening. Data are presented as means ± SD (*n* > 40). Dunnett’s test; *P < 0.05, ^§^*P* < 0.01. (**i**) Averaged gene expression of 8 selected GO terms for biological processes. (**j**) Compared to gelatin, known genes related to the development of cardiomyocytes were upregulated in PSC-CMs on laminin-511/521 such as cardiac marker genes (*Tnnt2* and *Actc1*), a transcription factor gene for cardiac gene expression (*Ankrd23*), a calcium handling gene (*Casq2*), and sarcomere genes (*Mybpc2*, *Mybpc3*, *Myh7*, and *Myl2*).

Finally, we examined if laminin-511/521 enhanced the functional and transcriptional maturation of PSC-CMs. We first examined the proportion of binuclear cells on laminin-511/521 and found that PSC-CMs cultured on laminin-511/521 showed a higher population of binuclear cells than gelatin (gelatin, 7.8%; laminin-511, 38.7%; laminin-521, 54.7%) (Fig. 5a). Next, we examined the localization of Connexin-43 (Cx43), a gap-junction protein, as it reflects cardiomyocyte maturation^33^. In embryonic cardiomyocytes, Cx43 is diffusely expressed in the cytoplasm. Then, in neonatal to young age, it localizes to the lateral side of cardiomyocytes. Once cardiomyocytes fully mature, Cx43 is mainly localized to the intercalated disc of cardiomyocytes. While PSC-CMs on gelatin expressed Cx43 weakly, PSC-CMs on laminin-511/521 strongly expressed Cx43 on lateral sides of the cells (Fig. 5b). These results suggested that the PSC-CMs were more mature and reached to the stage of neonatal or young age on laminin-511/521.

Cardiomyocytes turn glycolysis to fatty acid oxidation for energy production after birth. Thus, we next examined the mitochondria function of PSC-CMs using oxygen consumption rates (OCRs, Fig. 5c). On laminin-511/521, PSC-CMs displayed higher basal and maximal respiration levels, suggesting higher mitochondria activities than control PSC-CMs on gelatin (Fig. 5d-i-iv).

To further examine physiological changes as shown in Fig. 2, we performed calcium transients (Fig. 5e,f) and sarcomere shortening (Fig. 5g,h). For calcium transients, we compared gelatin versus PSC-CMs cultured on laminin-511/521. Representative traces are shown in Fig. 5e. The result showed that laminin-521 significantly reduced time to peak and also time to decay, while peak amplitude was higher in the PSC-CMs on laminin-511 (Fig. 5f). For sarcomere shortening, the representative traces are shown in Fig. 5g. We found that laminin-511/521 significantly increased percentage of sarcomere shortening (Fig. 5h). These results indicated that laminin-511/521 improved physiological properties of the PSC-CMs on them.

Finally, we also performed a transcriptome analysis. Compared to morphological or physiological changes we observed, sum expressions of specific GO terms were largely unchanged (Fig. 5i). However, detailed analysis revealed that sarcomere genes and maturation-related genes, identified in Fig. 3, tended to be upregulated in PSC-CMs on laminin-511/521 (Fig. 5j), suggesting that the effects of ECMs were through post-translational changes rather than transcriptional changes.

## Discussion

Generating mature cardiomyocytes from PSCs is crucial for heart disease modeling and drug discovery. However, to date, no method is available to assess PSC-CM maturation levels in a high-throughput fashion. Here, we generated a fluorescent Myom2-RFP reporter line that enables us to monitor cardiomyocyte maturation easily and rapidly.

Our data demonstrated that PSC-CMs derived from the fluorescent reporter mESCs incrementally expressed Myom2-RFP over time. RFP^+^ cardiomyocytes displayed more structural, morphological, physiological, and transcriptional maturation compared to RFP^−^ cells. Although RFP^+^ PSC-CMs are more mature, the cells are still not quite similar to adult cardiomyocytes even after 28 to 38 days of differentiation. Adult cardiomyocytes have highly organized sarcomeres, of which length is approximately 1.8–2.3 µm^34^. RFP^+^ cardiomyocytes also had an organized sarcomere structure with a sarcomere length of around 1.8 µm at day 28. RFP^+^ cardiomyocytes displayed a significantly higher calcium transient peak and faster decay time to baseline than RFP^−^ cells with sarcomere shortening at ~6.8%, though calcium transient of adult cardiomyocytes changes faster^12,15,35^. Moreover, cardiomyocytes transition from mononuclear to binuclear after birth (P7 to P10 in mouse)^36–38^. In mice, the proportion of binucleated cells present at birth and adult hearts are around 1.5% and 91.5%, respectively^36^. Notably, RFP^+^ cardiomyocytes became more binuclear (55.4% at day 28), while RFP^−^ cardiomyocytes remained mononuclear and only 10.8% was binuclear at day 28. Taken together, we estimated that RFP^+^ PSC-CMs are mature to a similar degree of cardiomyocytes at around P7. It appears that ECMs lead to more maturation but the PSC-CMs were still immature compared to adult cardiomyocytes. Since cardiomyocytes are exposed to microenvironments not only ECMs but also secreted factors and mechanical stress during heart development, combinations of those factors would lead to better cardiomyocyte maturation^39^.

To demonstrate if Myom2-RFP could be used for screening and identify a factor enhancing cardiomyocyte maturation, we examined the effects of ECMs and found laminin-511/521 E8 fragments strikingly enhanced cardiomyocyte maturation in all aspects we examined. E8 fragments are the minimal structure of laminin composed of the C-terminal regions of the α, β and γ chains with retained interactions with integrins, and have a higher adhesion affinity for cells than intact laminin^32^. ECM-integrin interactions regulate several cellular responses, including adhesion, expansion, and differentiation^32,40^. Integrins are heterodimeric transmembrane receptors consisting of α and β subunits, and at least eighteen α and eight β subunits are known in mammals^41,42^. Their combinations give rise to a variety of heterodimers, which differ in their ligand specificities. For instance, integrin α3β1 and α6β4 are the major receptors mediating cell adhesion on laminin-332 and laminin-511, whereas integrin α6β1 has a preference for laminin-111, laminin-332, and laminin-511/521^43^. Thus, these integrins can be potential partners of laminin-511/521 to mediate cardiomyocyte maturation, though the molecular mechanism for cardiomyocyte maturation mediated by laminin-integrin remains to be elucidated in further study.

In summary, we have developed a Myom2-RFP reporter line, which allows examination cardiomyocyte maturation easily and rapidly. Most importantly, this reporter will be a valuable tool for screening interested external stimuli of interest such as ECMs, hormones, and even for optimizing maturation strategies to robustly enhance cardiomyocyte maturation. We believe that this fluorescent reporter line will facilitate the utility of PSC-CMs for heart disease modeling and new therapeutic drug screening in the future.

## Experimental Procedures

### Mouse experiments

All animal experiments reported here were approved by the Institutional Animal Care and Concern Committee at Jichi Medical University. Animal cares and all experiments were performed under the committee’s guidelines. We obtained mouse hearts at the ages shown in the figure or figure legend from C57BL/6 J purchased from SLC Japan (Shizuoka, Japan).

### mESC maintenance

syNP4 cells^6^ were used as the parental line in this study and maintained in 2i medium^44^ containing Glasgow minimum essential medium (Sigma) with 10% fetal bovine serum (FBS, Moregate), 1,000 U/ml LIF, 0.1 mM 2-mercaptoethanol (Thermo Fisher Scientific), 3 μM CHIR99021, 1 μM PD0325901, Glutamax (Thermo Fisher Scientific), sodium pyruvate (Thermo Fisher Scientific), and MEM non-essential amino acids (Thermo Fisher Scientific), on gelatin-coated tissue culture plate. Cells were routinely passaged every two to three days.

### Generation of Knock-in mESCs

Single-guide RNA (sgRNA) targeting 3′ end of *Myom2* (5′-GCTTCCACCTCATCTGATTA + AGG-3′; the last TAA is the stop codon of *Myom2*) was designed and cloned to px330 as previously described^45^. Approximately 1 kb homology arms of both sides from the targeting site were cloned. TagRFP and blasticidin-resistance cassette were inserted to replace the stop codon of Myom2 and PAM sequence (Fig. 1d-ii). TagRFP was designed to be in-frame to endogenous Myom2. px330 with Myom2 sgRNA and the targeting vector were co-transfected to syNP4 cells. Blasticidin-resistant clones were screened with PCR using primer combinations of 5F2/TR and Blast/Downstream (Fig. 1d-iii). Site-specific integration was confirmed by sequencing. After efficient cardiac differentiation as syNP4 and RFP expression in a prolonged culture was confirmed, CAG-Flpe-IRES-Puro was then transfected to a clone (SMM18) to remove the blasticidin-resistance cassette. A short-term treatment of puromycin (for one day) was conducted to enrich transfected cells. The cassette removal was confirmed by PCR with primer combinations of 3F1/Downstream and Blast/Downstream. We also confirmed cells were sensitive to blasticidin and puromycin at the undifferentiated stage. We again confirmed clones differentiated well to puromycin-resistant cardiomyocytes and RFP expression after a prolonged culture. Primer sequences used in this study were: 5F2, 5′-GTCACAGGGACATAGGCACTT-3′; TR, 5′-GATGTGCACTTGAAGTGGTG-3′; Blast, 5′-AAAAGCCTCCTCACTACTTCTGG-3′; Downstream, 5′-GAAGGGTACTTAACCCAGGAACC-3′; 3F1, 5′-GAGGACTCGGGCAAGTACAG-3′.

### Cardiomyocyte differentiation

Cardiac differentiation was conducted as described previously by our group^12^. Briefly, SMM18 or SMMB2 cells were suspended in serum-free differentiation medium (SFD)^7^ containing Iscove’s modified Dulbecco’s medium (Thermo Fisher Scientific) and F12 medium (Thermo Fisher Scientific), supplemented with B27 without retinoic acid (Thermo Fisher Scientific), N2 supplement (Thermo Fisher Scientific), Glutamax, ascorbic acid (Wako), and 1-thioglycerol for 2 days. Then, cells were primed toward mesoderm by culturing with growth factors including activin-A (R&D Systems), BMP4 (R&D Systems), and VEGF (Wako) from day 2 to day 4. At day 4, cells were plated and induced to the cardiac lineage using bFGF (Wako), FGF10 (R&D Systems), and VEGF. After 7 days, beating-cells were observed. To enrich cardiomyocyte yields, fresh medium-containing puromycin was fed to the cells at day 7 and cultured for 3 days. Following antibiotic selection, more than 90% of cells expressing the cardiomyocyte marker, cardiac troponin T (cTNT), were obtained. The purified cardiomyocytes were used for further analyses. For the long-term culture, we plated the cardiomyocytes at day 10 and cultured in the SFD medium up to day 38 of differentiation.

### Immunostaining

For hearts, tissues were embedded and directly frozen in optimal cutting temperature (OCT) compound (Tissue-Tek, Sakura) and cut on a Leica Cryostat (4 μm). Sections were washed with PBS before fixed in 4% paraformaldehyde (PFA, Wako) for 30 minutes at 4 °C. Then sections were washed and permeabilized using 0.1% Triton X-100 (Amersham Biosciences) in PBS for 15 minutes at room temperature. After permeabilization, cells were blocked with 3% bovine serum albumin followed by incubation with anti-Myom2 polyclonal antibody (LS-B9842, 1:100, LifeSpan BioSciences) and anti-α-actinin monoclonal antibody (EA-53, 1:100, Sigma-Aldrich) overnight at 4 °C. Sections were then washed with PBS containing 0.2% Tween-20 (Nacalai Tesque), and stained with secondary antibodies, anti-rabbit IgG (1:500, Thermo Fisher Scientific) and anti-mouse IgG (1:500, Thermo Fisher Scientific), conjugated with Alexa Fluor Plus 555 and 488, respectively. DAPI (Dojindo Laboratories) was used for nuclei staining. The slides were mounted in VECTASHIELD Antifade Mounting Medium (Vector Laboratories).

For PSC-CMs, the cells cultured in CellCarrier 96-well black polystyrene microplates (PerkinElmer) were fixed with 4% PFA for overnight. After that, cells were washed with PBS and permeabilized using 0.2% Triton X-100 in PBS for 15 minutes at room temperature. Then, cells were blocked with 2% FBS in PBS followed by incubation with anti-α-actinin antibody (1:500) or anti-cardiac troponin T monoclonal antibody (13–11, 1:500, Thermo Fisher Scientific), and anti-tRFP (AB233, 1:500, Evrogen) for overnight at 4 °C. Cells were washed and stained with secondary antibodies, anti-mouse IgG (1:500, Thermo Fisher Scientific) and anti-rabbit IgG (1:500, Thermo Fisher Scientific), conjugated with Alexa Fluor 488 and 555, respectively. Nuclei were stained with DAPI solution.

Immunofluorescent images were collected by a confocal laser scanning microscope (Olympus FluoView FV1200) or an inverted fluorescence microscope (Olympus IX83). Sarcomere length, cell size, circularity, perimeter, as well as cell geometry, were analyzed by ImageJ software.

### Quantification of sarcomere alignment index

The immunofluorescent images for α-actinin were transformed into frequency spectrum images for the evaluation of the distributions of orientation frequencies by OrientationJ, an ImageJ plugin, for directional analysis. To calculate the sarcomere alignment index, we used a formula as follows:

$$Alignment\,Index=\frac{\eta /\eta +\omega }{{\eta }_{IR}/{\eta }_{IR}+{\omega }_{IR}}$$

η is a frequency within 20° from peak angles at −90° and 90°, while η + ω is a frequency of total distribution. Thus, η/η + ω is the fraction of sarcomeres aligned vertically to the longitudinal axis of a cardiomyocyte. η_IR_ and η_IR_ + ω_IR_ are the same frequencies of the distributions in an ideal random histogram (Supplementary Fig. S2c). This formula was applied from a previous report, in which the formula was used to evaluate fibroblast alignment^46^. A high value of alignment index indicates a well-organized sarcomere, whereas a low value represents a disorganized sarcomere or random alignment.

### Calcium transients and sarcomere shortening

To determine intracellular calcium transients, PSC-CMs were cultured for 28 days in a glass-bottom 24-well plate (MatTek Corporation). Then, cells were washed with PBS and loaded with Calbryte 520-AM (AAT Bioquest) in Tyrode’s solution (containing 140 mM NaCl, 5.4 mM KCl, 0.5 mM MgCl_2_, 0.33 mM NaH_2_PO_4_, 2 mM CaCl_2_, 5 mM HEPES, and 11 mM D-glucose, pH adjusted to 7.4 with NaOH) for 30 minutes. Cells were evoked by electrical field stimulation at 1 Hz (C-Pace, IonOptics). Intracellular calcium transients were recorded with a 40x objective lens, 10 msec exposure and 20 msec interval by an inverted fluorescence microscope (Olympus IX83 with ORCA-Flash4.0 V3). ImageJ was used to quantify intracellular calcium transients as described in the previous study^47^. For sarcomere shortening, cell contraction was recorded continuously with time-lapse videos for live-cell imaging. The time-lapse recordings were then analyzed by SarcOptiM for the ImageJ as explained in the previous study^48^.

### Mitochondrial activity assay

Mitochondria function was analyzed with Seahorse XF96 extracellular flux analyzer. Seahorse XF96 microplate was coated with 0.1% gelatin or ECMs. At day 10 of cardiac differentiation, the PSC-CMs were seeded onto the plate with a density of 50,000 cells/well. The cells were cultured until day 38 before starting the assay. The culture medium was changed for base medium (Seahorse XF RPMI medium supplemented with 1 mM pyruvate, 2 mM glutamine, and 25 mM glucose) for 1 hour before running assay and during measurement. Selective inhibitors were sequentially injected during the measurements at the final concentrations of 3 μM oligomycin (an inhibitor for complex V [ATPase] of mitochondrial electron transport chain [METC]), 0.5 μM carbonyl cyanide-*p*-trifluoromethoxyphenylhydrazone (FCCP, as a mitochondrial oxidative phosphorylation uncoupler), and 3 μM rotenone (an inhibitor for complex I of METC) and antimycin A (an inhibitor for complex III of METC). Basal respiration was represented by oxygen consumption rate (OCR) before applying oligomycin. ATP-linked respiration was represented by the oligomycin-sensitivity respiration rate, while proton leak was calculated by the difference between oligomycin and rotenone/antimycin A rates. Maximal mitochondrial respiration was the response to FCCP.

### Flow cytometry

PSC-CMs generated from the Myom2-RFP ESC line were dissociated to single cells by treatment with TrypLE (Thermo Fisher Scientific) for 10 minutes, at 37 °C. After washing with PBS, the cells were suspended with 2% FBS in PBS containing DAPI (1:2000). Measurements of the proportion of RFP^+^ and RFP intensity and cell-sorting were performed with SH800 (SONY).

### RNA sequencing

Total RNA was extracted from the PSC-CMs at different time points of differentiation according to the manufacturer’s protocol for Direct-zol RNA extraction kit (Zymo Research), whereas RNA from mouse hearts was isolated by standard acid guanidinium thiocyanate-phenol-chloroform extraction using TRIzol. The quantity of the RNA samples was determined by Qubit 4 Fluorometer (Thermo Fisher Scientific). cDNA was prepared by QuantSeq. 3′ mRNA-Seq library prep kit FW for Illumina (Lexogen) according to the manufacturer’s protocol using ~500 ng total RNA per samples. Library concentrations and size-distribution were then confirmed by Qubit 4 Fluorometer and Agilent 2100 Bioanalyzer (Agilent Technologies), respectively. cDNA library was pooled from each cDNA sample and sequenced by the Illumina NextSeq (75 cycles, single-end). Adapter and quality trimming was performed with BBDuk, then the trimmed reads were mapped to the GRCm38 mouse genome using STAR RNA-seq aligner^49^. To count the mapped read, featureCounts was used^50^. The read counts were normalized to transcript per million to show gene expression levels and/or compare expression levels between genes. To compare expression levels between samples, the read counts were normalized to regularized log (rlog) using DEseq. 2^51^. To perform GO analysis, enrichGO function in clusterProfiler was used^52^.

### Statistical analysis

Data are presented as mean ± standard deviation (SD) for at least three replicate samples. Student’s *t*-test, Dennett’s test, chi-square test, or one-way ANOVA were used where appropriate as described in legends. All of the statistical analysis was performed using R statistical software. A *P*-value of less than 0.05 was considered significant.

## Supplementary information

Supplementary information.
