# Supplementary material for: A Novel Fluorescent Reporter System Identifies Laminin-511/521 as Potent Regulators of Cardiomyocyte Maturation
Source: Sci Rep. 2020 Mar 6;10:4249. doi: 10.1038/s41598-020-61163-3 (PMC7060274; doi:10.1038/s41598-020-61163-3)
Supplement: Supplementary file 1 — Supplementary information. [file 41598_2020_61163_MOESM1_ESM.pdf]

A Novel Fluorescent Reporter System Identifies Laminin-511/521 as Potent Regulators of Cardiomyocyte Maturation

Nawin Chanthra[1], Tomoyuki Abe[1], Matthew Miyamoto[2,3], Kiyotoshi Sekiguchi[4], Chulan Kwon[2,3], Yutaka Hanazono[1,5], Hideki Uosaki[1,5,*]

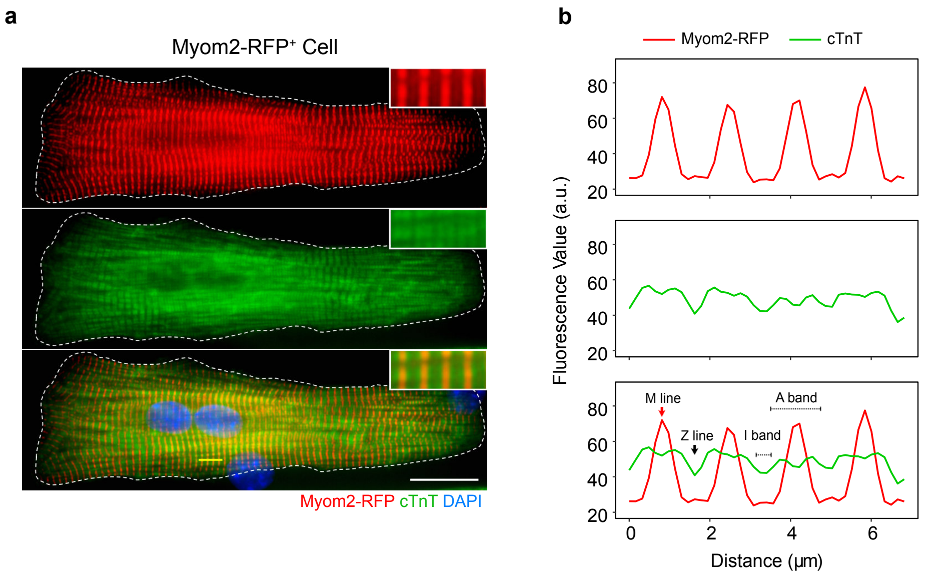

Supplementary Figure S1. Localizations of Myom2 and cTnT in sarcomere structure. (**a**) Representative fluorescence images of RFP^+ cardiomyocyte. Yellow lines indicate line-scan regions shown in (b). The regions around the lines are shown in inset. Myom2-RFP (red); cTnT (green); Nuclei (blue). Scale bar, 20 μm. (**b**) Corresponding line scans for Myom2-RFP and cTnT localizations.

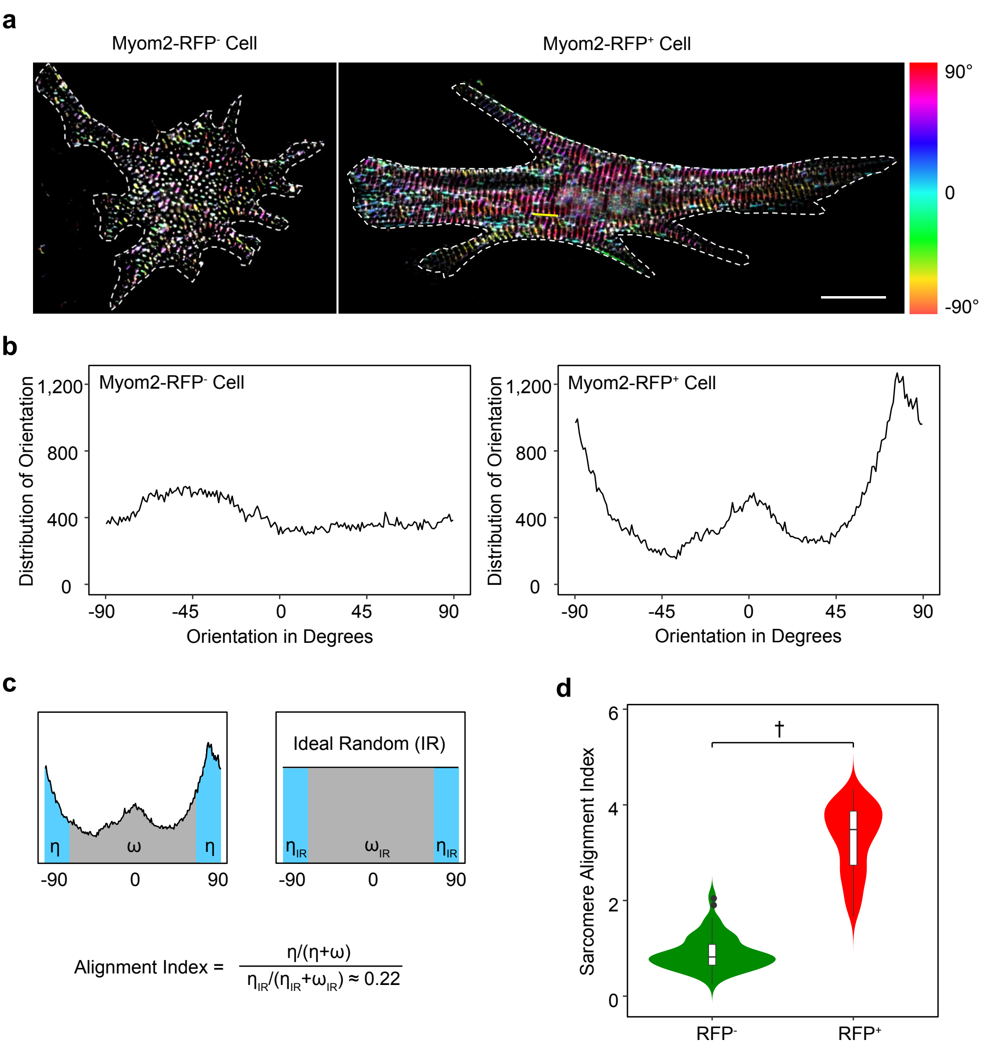

Supplementary Figure S2. Comparison of sarcomere alignment between RFP$^-$ and RFP$^+$ cardiomyocytes. (**a**) The color-coded representations of the α-actinin orientation in both RFP$^-$ and RFP$^+$ cardiomyocytes. Scale bar, 20 μm. (**b**) The corresponding distributions of orientation shown in (a). (**c**) From the orientation distribution, sarcomere alignment index was calculated by sum of frequencies within 20° from peak angles at -90° and 90° (η, blue area) against total distribution frequency ($\eta+\omega$). Then, this ratio was normalized to that of an ideally random frequency distribution ($\eta_{IR}/(\eta_{IR} + \omega_{IR})$), ~0.22. (**d**) RFP$^+$ cardiomyocytes showed higher sarcomere alignment index than RFP$^-$ cardiomyocytes (n > 65, from three different cardiac differentiation runs). Violin plots are explained in Fig. 2. Student’s t test; † P < 0.0001.

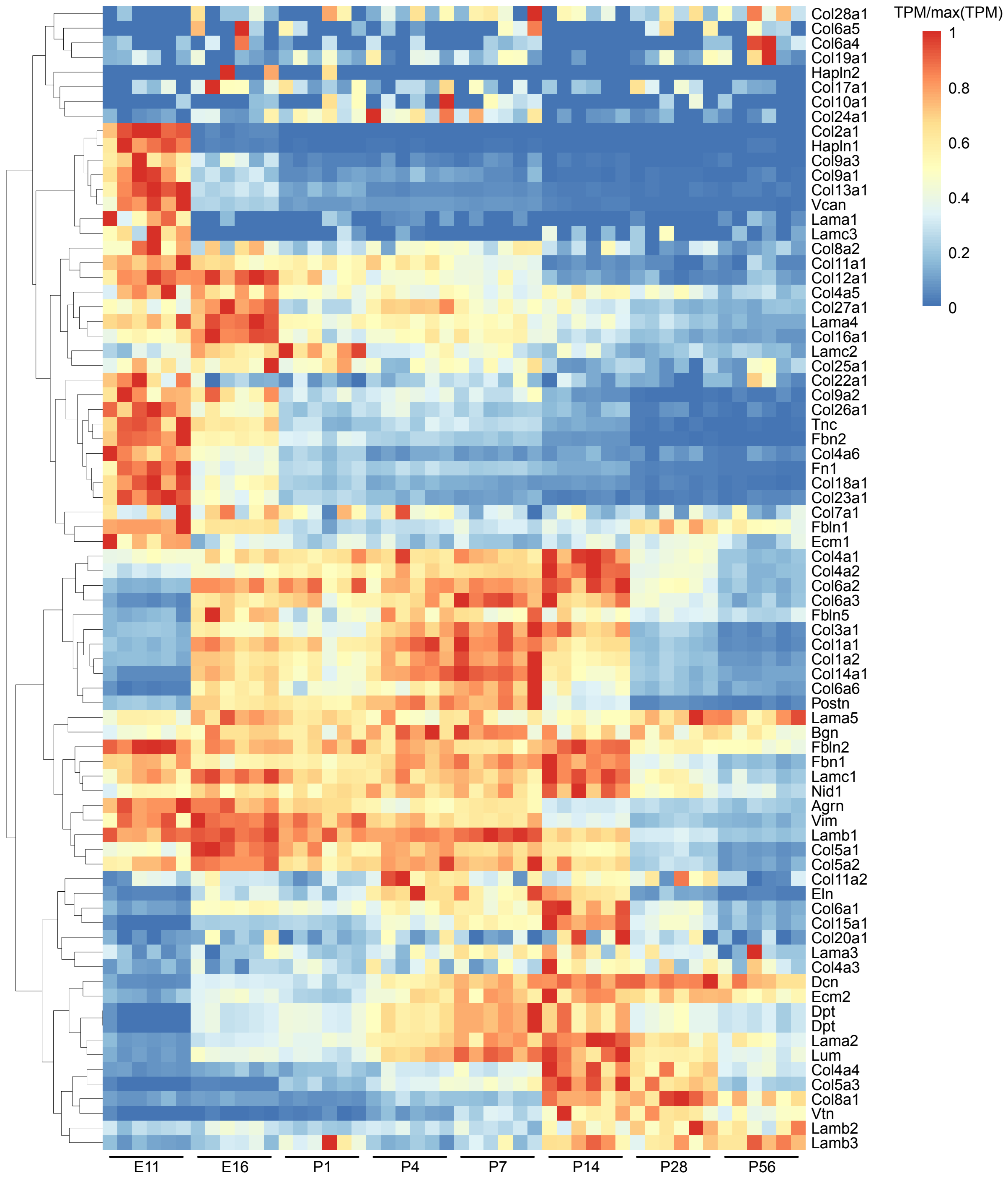

Supplementary Figure S3. A heatmap of ECM expressions during heart development (E11 to P56). Colors are coded on the red-to-blue scale of normalized transcripts per million reads (TPM) divided by the highest expression level (TPM/max: high expression, red; low expression, blue).

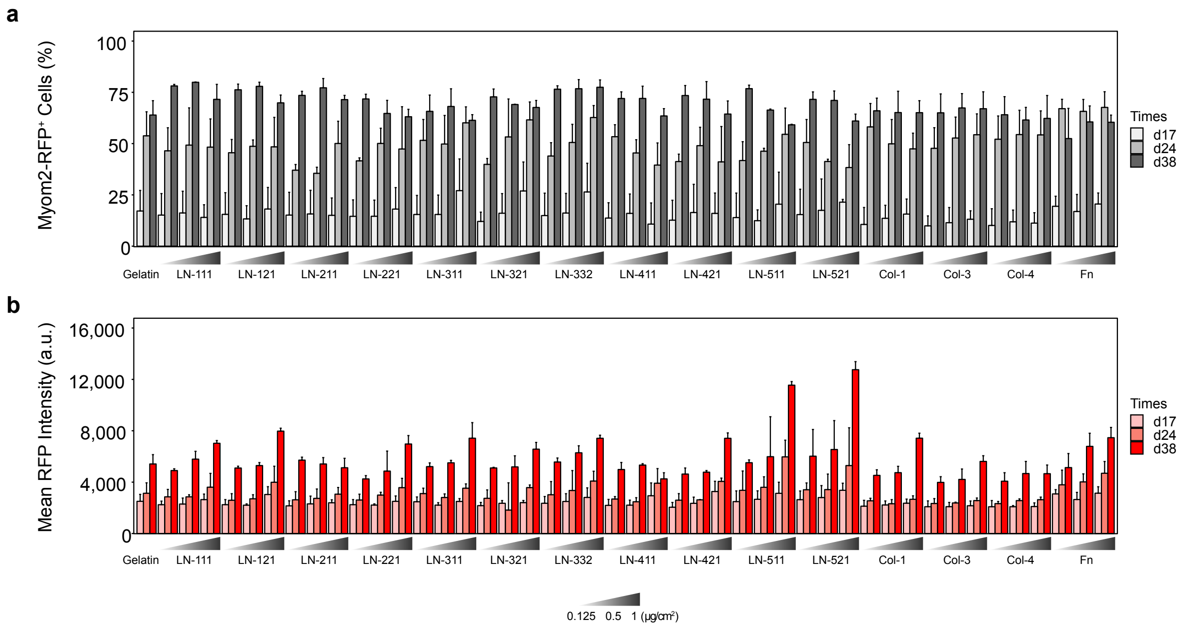

Supplementary Figure S4. ECMs increased RFP intensity as a dose-dependent manner. (**a**) Percentages of Myom2-RFP$^+$ cardiomyocyte and (**b**) RFP intensity at day 17, 24, and 38. ECM concentration are shown at 0.125, 0.5, and 1.0 μg/cm^2. Data are presented as means ± SD (*n* = 3). Fluorescence intensities are presented as arbitrary units (a.u.).
